# Stimulus-driven updating of long-term context memories in visual search

**DOI:** 10.1007/s00426-021-01474-w

**Published:** 2021-01-26

**Authors:** Markus Conci, Martina Zellin

**Affiliations:** Allgemeine und Experimentelle Psychologie, Department Psychologie, Ludwig-Maximilians-Universität München, Leopoldstr. 13, 80802 Munich, Germany

## Abstract

Visual search for a target is faster when the spatial layout of nontarget items is repeatedly encountered, illustrating that learned contextual invariances can improve attentional selection (contextual cueing). This type of contextual learning is usually relatively efficient, but relocating the target to an unexpected location (within otherwise unchanged layouts) typically abolishes contextual cueing. Here, we explored whether bottom-up attentional guidance can mediate the efficient contextual adaptation after the change. Two experiments presented an initial learning phase, followed by a subsequent relocation phase that introduced target location changes. This location change was accompanied by transient attention-guiding signals that either up-modulated the changed target location (Experiment 1), or which provided an inhibitory tag to down-modulate the initial target location (Experiment 2). The results from these two experiments showed reliable contextual cueing both before and after the target location change. By contrast, an additional control experiment (Experiment 3) that did not present any attention-guiding signals together with the changed target showed no reliable cueing in the relocation phase, thus replicating previous findings. This pattern of results suggests that attentional guidance (by transient stimulus-driven facilitatory and inhibitory signals) enhances the flexibility of long-term contextual learning.

## Introduction

Attentional orienting in visual search is often supported by statistical regularities in the natural environment (Bar, 2004; Oliva & Torralba, 2007, for reviews). For instance, visual search for a target object (e.g., a loaf of bread) is faster when it is associated with a related scene context (e.g., a kitchen scene) rather than with an unrelated context (e.g., a front yard, Palmer, 1975; see also Davenport & Potter, 2004; Conci & Müller, 2014; Draschkow & Võ, 2017). However, even though objects may occur in some environments but not in others, thus providing predictive cues for behaviour, changes to an object might nevertheless occur at any time. If observers already have a memory representation of the contextual information that undergoes some kind of change, learned associations would have to be updated to achieve a continued benefit from statistical regularities for efficient orienting in the environment (see Conci, Zellin, & Müller, 2012). For example, if the loaf of bread is relocated from its usual location to a new location in a familiar kitchen scene, then previously established contextual associations would have to be relearned, or adapted, to include the new target position. In this study, we examined whether redirecting attention can speed such updating, to incorporate the changed target in a given learned memory representation efficiently.

**Fig. 1:**
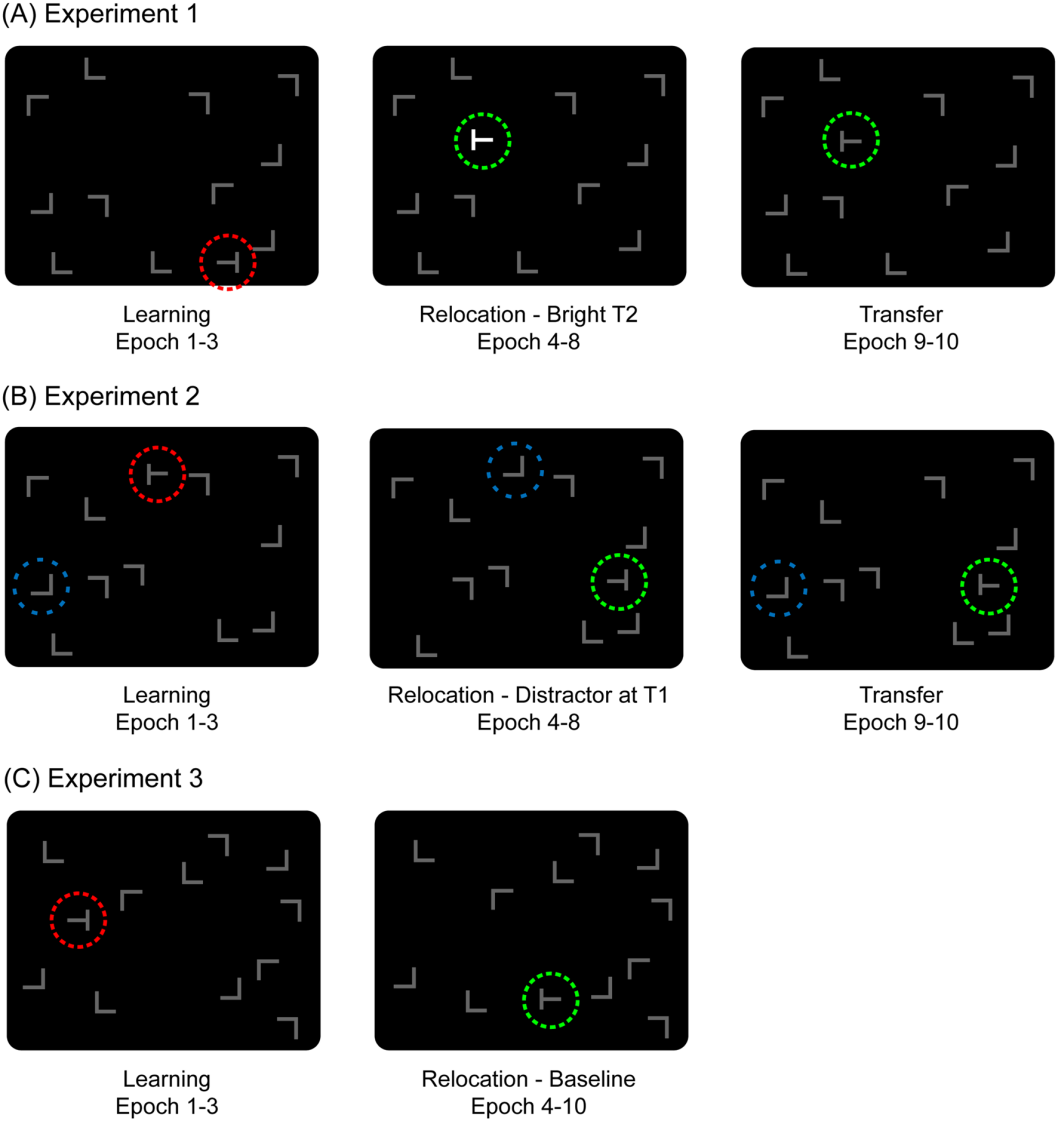
Examples of old-context displays illustrating the changes across experimental phases in Experiments 1 (**a**), 2 (**b**) and 3 (**c**). Search displays were initially paired with fixed, unique target locations in the learning phase (T1, red circle). Subsequently, in the relocation phase, targets were repeatedly presented at novel, previously empty positions (T2, green circle). The change in target location was accompanied by an attentional guidance signal, which either constituted **a** a transient T2 brightness enhancement, or **b** a repositioning of a distractor L to the location of the previous T1 target (blue circle). Experiment 3 **c** served as a baseline, and was identical to Experiments 1 and 2, except that the change of the target location was not accompanied by a concurrent guidance signal. A final transfer phase (in Experiments 1 and 2) then presented the novel T2 target without additional guidance signals. The coloured circles were not shown in the actual displays, but illustrate the location changes of the target (and of the nontarget in Experiment 2)

Previous studies have shown that observers implicitly learn spatial relations between an invariant context of nontargets and a given target location, which facilitates visual search for that target location on subsequent encounters (contextual cueing; Chun & Jiang, 1998). In a typical experiment, observers search for a target letter ‘T’ amongst a configuration of eleven nontarget ‘Ls’ (see Fig. 1) and indicate the orientation of the target (left vs. right). Unknown to the observers, some of the search displays are repeatedly presented with invariant spatial configurations of the target and nontargets (old contexts) throughout the experiment. Results show faster response times (RTs) to old contexts than search in a condition of randomly generated, new contexts. This ‘contextual-cueing effect’ indicates that attention is (incidentally) guided to the target location by the learned associations in old contexts. Thus, the repeated context provides some predictive cues that are learned and thereby help to detect the target more quickly on future occasions (Zinchenko, Conci, Müller, & Geyer, 2018).

While changes in the objects’ identities do not seem to affect *spatial* contextual cueing (Chun & Jiang, 1998), a permanent change of the target location typically leads to a rather pronounced reduction of contextual cueing (Annac, Conci, Müller, & Geyer, 2017; Conci, Sun, & Müller, 2011; Conci & Müller, 2012; Makovski & Jiang, 2010; Manginelli & Pollmann, 2009; Zellin, Conci, von Mühlenen, & Müller, 2013a). For example, in the study by Manginelli and Pollmann (2009) repeated spatial contexts were paired with unique target locations and repeatedly presented in an initial learning phase, thus revealing a typical contextual-cueing effect. Subsequently, targets were relocated within their spatial contexts, and this change of the target location in turn reduced contextual cueing. Even though targets appeared repeatedly at the new locations, contextual cueing did not recover (instead, the repeated context still provided a bias to the initially learned target location, see Zellin et al., 2013a). This general pattern of results was also replicated in a recent meta-analysis (Annac et al., 2017) that combined the results from 85 participants pooled across 7 previous (own) experiments. These combined results revealed a reliable 131-ms RT benefit for old contexts (relative to new layouts) in the initial learning phase, which was, however, reduced (by 97%) to a non-reliable 4-ms cueing effect after the target location change. This indicates that contextual cueing is initially rather efficient to extract invariant statistical regularities. Yet, a subsequent change of the target location within an otherwise unchanged nontarget layout largely abolishes contextual cueing. A reliable benefit for relocated targets was only evident when observers completed an intensive training with relocated targets, where observers were trained on 4 consecutive days with the changed target location (Zellin, von Mühlenen, Müller, & Conci, 2014). Such extended training with the changed target resulted eventually in contextual cueing for both target locations: observers actually learned both the initial target locations and the relocated targets. However, efficient relearning (if present at all) is usually rather slow and effortful to eventually incorporate associations between a single context and multiple (i.e., two) target locations that are associated with it.

When observers are faced with a changed target location in otherwise repeated contexts, of which they already maintain a memory representation, old associations are likely to intrude and proactively interfere with learning of changed associations (Zellin et al., 2013a; see also Lustig & Hasher, 2001; Mednick, Makovski, Cai, & Jiang, 2009; Anderson & Neely, 1996, for an overview). For instance, it has been shown that old contexts guide visual attention to initially learned target locations (Johnson, Woodman, Braun, & Luck, 2007; Ogawa, Takeda & Kumada, 2007). However, after a change of the target location, the old context still provides an automatic attentional bias signal towards the original target location, thereby interfering with efficient relearing (Zinchenko, Conci, Töllner, Müller, & Geyer, 2020). This indicates that once established, a persisting association leads to some form of ‘misguidance’ (Manginelli & Pollmann, 2009) to a single, initially learned target location (Zellin, Conci, von Mühlenen, & Müller, 2011) that probably results from a lack of updating predictions in attentional control structures in frontal cortex (Pollmann & Manginelli, 2009; Zinchenko, Conci, Taylor, Müller, & Geyer, 2019). In the current study we therefore aimed to facilitate the updating of a previously established contextual cue—by providing bottom-up signals that can potentially help to incorporate the changed target in an already—established memory representation.

Bottom-up signals that guide attention can, for example, be revealed in cases where an object is more salient than the surrounding objects. For instance, salient items may increase the attentional priority of that item and thereby reveal an influence upon performance (e.g., Theeuwes, 1992; Horstmann, 2002; von Mühlenen & Conci, 2016; Ruthruff, Faulks, Maxwell, & Gaseplin, 2020). Moreover, when observers performed a contextual cueing experiment with displays that contained a salient red nontarget item (among otherwise uniform grey items), overall response latencies were increased and the mean contextual-cueing effects were reduced in comparison to displays that presented unicoloured search items (Conci & von Mühlenen, 2009). In addition, presenting a target among a group of items that appear in a salient colour results in a larger contextual-cueing effect, as compared to a situation where the target is presented in a group of non-salient items (Conci & von Mühlenen, 2011). This indicates that saliency may—at least to a certain extent—attract attention in a stimulus-driven manner, and thus modulate the efficiency of contextual learning.

Contextual learning can also, in itself, modulate the priority of the individual search items. For instance, it has been shown that contextual cueing leads to a facilitation of the invariant target locations (Brady & Chun, 2007; Geyer, Zehetleitner, & Müller, 2010). Ogawa, Kumada and Takeda (2007) in turn showed that contextual cueing also leads to the inhibition of nontarget locations, that is, nontargets in old contexts are more effectively “ignored” (see also Makovski & Jiang, 2010). An inhibitory bias towards nontarget items may also be revealed in both efficient and inefficient searches (e.g. Klein, 1988; Müller, von Mühlenen, & Geyer, 2007) irrespective of statistical learning. For instance, attentional guidance in visual search has been assumed to be driven in part by some inhibitory mechanism that tags nontarget locations to prevent that these irrelevant items are repeatedly (re-)visited. Together, these findings indicate that basic visual search (and contextual cueing) may be affected by two orthogonal operations, namely from the facilitation of the target location, and from the inhibition of (irrelevant) nontargets, thereby optimizing search performance.

In the present study, we tested whether the bottom-up modulation of attention by facilitatory and inhibitory signals can facilitate the updating of learned contextual associations after a change of the target location. Previous studies have shown that observers need time-consuming training to adapt contextual associations to changed target locations (Zellin et al., 2014). This lack of an efficient contextual adaptation presumably occurs because observers are not necessarily aware that they have learned a certain environmental regularity, and therefore, the previously learned (old) context still biases attention towards the initial target locations in an automatic manner (Zinchenko et al., 2020), thus constraining efficient relearning. Here, we tested whether guidance to initial target locations can be reduced by providing bottom-up signals in the search displays that improve the adaptation towards changed target locations.

We present three experiments that all started with a learning phase, in which invariant context-target configurations were repeatedly presented, thus establishing a typical contextual-cueing effect (see Fig. 1). Subsequently, targets were relocated and repeatedly presented at new, previously empty, locations within their respective contexts (e.g., Manginelli & Pollmann, 2009; Zellin et al., 2013a). To potentially facilitate relearning during this relocation phase, in Experiment 1, relocated targets were presented with increased brightness to make the changed target locations more salient than the surrounding context of nontargets (Fig. 1a). Such an increase in salience should guide attention to changed target locations more efficiently (e.g. Itti & Koch, 2001). In Experiment 2, rather than increasing the salience of the changed target, a (non-salient) nontarget was placed at the initial target location (Fig. 1b). Task-irrelevant nontargets are typically inhibited (e.g., Klein, 1988) to avoid that the nontarget’s location is repeatedly re-visited. Now, presenting a nontarget at the initial target location should therefore lead to an inhibitory signal, and this should increase the incentive to avoid this location and rather incorporate the relocated target in the existing memory representation. The last phase of both experiments (transfer) tested whether the attentional modulation in the relocation phases would result in successful, sustained adaptation of contextual associations to relocated targets. For instance, displays were presented with the relocated targets, but with the same salience as nontargets (Experiment 1), or without the inhibitory nontarget at the initial target location (Experiment 2). Finally, Experiment 3 was performed to obtain a baseline measure, to replicate the typical lack of adaptation subsequent to a target location change in an experiment that was directly comparable to Experiments 1 and 2 (except for the additional, attention-guiding signals in the relocation phase; Fig. 1c). Thus, if the attentional modulation towards the relocated targets (in Experiments 1 and 2) was successful, then the contextual associations from the learning phase should increase the chances for the efficient updating after the change (but such updating should not be evident in Experiment 3 where no attention-guiding signal was presented).

## Experiment 1

The aim of Experiment 1 was to facilitate the adaptation towards a previously learned but subsequently changed target location by transiently increasing the salience of the changed target relative to the surrounding context of nontargets. Salient objects in a visual search display typically attract attention (Itti & Koch, 2001), and as a result, modulate contextual cueing (Conci & von Mühlenen, 2009). A salient target should therefore lead to more effective guidance to the salient, relocated targets in Experiment 1, and this attention-guiding signal should in turn facilitate the updating of the memory representation to incorporate the changed target location.

### Methods

#### Participants

In Experiment 1, we tested a sample of 18 adults (10 women, mean age: 29.7 years). All observers reported normal or corrected-to-normal visual acuity and were right-handed. They received either payment (10 €) or course credits for their participation. The study was approved by the ethics committee of the Department of Psychology at LMU Munich, and all participants provided written informed consent prior to the experiment. As in several previous studies (e.g., Annac et al., 2017; Conci et al., 2011; Kunar & Wolfe, 2011; Olson, Chun, & Allison, 2001; Zellin et al., 2014), only observers who showed contextual cueing [RT(new)-RT(old)] larger than zero in the initial learning phase were included in the analysis. Given that the current study aimed to investigate how a change in target locations affects *previously acquired* contextual associations, observers who failed to learn the repeated contextual layouts in the first part of the experiment had to be excluded. Overall, a total of 32 observers needed to be tested in Experiment 1, to obtain a sample of 18 observers that revealed larger-than-zero cueing effect in the initial part of the experiment. The same procedure was also adopted in the subsequent experiments.

The estimation of the required sample sizes was informed by previous contextual-cueing studies that also compared contextual learning and (the lack of) adaptation across sequential phases (e.g., Zellin et al., 2013a, 2014; Zinchenko et al., 2019). On the basis of the number of participants tested in and statistical measures provided by these studies, a sample size of 12–14 participants suffices to detect a lack-of-adaptation effect with a power of 0.8 in a single experiment. That is, on the basis of these previous studies, one would expect that the cueing effect should vanish after the target-location change, which would be evidenced by a significant two-way context by phase interaction (alongside with a corresponding reduction in contextual cueing). We further increased our sample to N = 18 observers (“learners”) to ensure sufficient statistical power in our analyses.

#### Apparatus and stimuli

Stimulus presentation and response collection was controlled by an IBM-PC compatible computer using Matlab Routines and Psychophysics Toolbox extensions (Brainard, 1997; Pelli, 1997). A standard mouse was used as the response device. Stimuli subtended 0.7° × 0.7° of visual angle and were presented in dim (8.5 cd/m^2^) or bright (178 cd/m^2^) grey against a black background (0.2 cd/m^2^) on a 17″ CRT monitor. Search displays consisted of 12 items, one of which was a T-shaped target rotated randomly by 90° either to the left or right. The 11 remaining items were L-shaped nontargets rotated randomly in one of four orthogonal orientations. Search displays were generated by placing the target and nontargets randomly in the cells of a 6 × 8 matrix, with an individual cell size of 2.5° × 2.5°. Nontargets were jittered horizontally and vertically in steps of 0.1°, within a range of ± 0.6°. Example search displays are shown in Fig. 1a. Observers were seated in a dimly lit room with an unrestrained viewing distance of approximately 57 cm from the computer screen.

#### Trial sequence

Each trial started with the presentation of a fixation cross at the centre of the screen for 500 ms. Then, a search display appeared and remained visible until participants made a speeded response by pressing one of two mouse buttons (with the left- and right-hand index finger, respectively). Observers were instructed to search for the rotated ‘T’ and decide as quickly and accurately as possible whether the stem of the T was pointing to the left or the right. In case of a response error, a minus sign appeared on the screen for 1000 ms. An inter-stimulus interval of 1000 ms separated one trial from the next.

#### Design and procedure

Experiment 1 used a repeated-measures design, with the (within-subject) factors Context (old, new) and Epoch (1–10). A set of 12 old-context displays with an invariant arrangement of nontarget items was generated for each observer and repeated throughout the experiment. For new contexts, the configuration of nontarget items was generated randomly on each trial. Each old- and new-context display was paired with two different target locations. Different sets of target locations were selected for old and new contexts, such that overall, 48 possible target locations were used. These 48 possible locations (arranged in a 6 × 8 matrix) in the search display were divided into 3 regions (i.e., rings around fixation), where 8 locations in the display centre would directly surround the fixation cross, another 16 locations would be presented with an intermediate distance from fixation, and the remaining 24 locations would be presented with the largest distance from central fixation. Now, for each of the 2 distinct target locations in each of the old/new context display sets, 2 target locations would be randomly selected from the inner region, 4 target locations from the intermediate region and 6 from the outer display region. This measure ensured that there was no systematic difference in eccentricity across conditions (see also Jiang & Sisk, 2019), while also presenting unique target locations for each individual display (i.e., to avoid a possible interaction due to global statistical learning, see Zellin, von Mühlenen, Müller, & Conci, 2013b; Wang et al., 2020). The orientation of the target was random on each trial, whereas nontarget orientations were constant in old contexts. The second factor Epoch divided the experiment into ten equally sized consecutive bins (each bin consisted of 120 trials averaging trials from 5 consecutive blocks).

The experiments started with a practice block of 24 randomly generated displays. All subsequent experimental blocks consisted of 24 trials, 12 with old- and 12 with new-context displays presented in random order.

An example sequence of the three experimental phases in Experiment 1 is presented in Fig. 1a. Displays were presented with initial target locations (T1) in the first 15 blocks (aggregated into 3 epochs; initial learning phase). In 25 subsequent blocks (epochs 4–8; relocation phase) displays were presented with relocated targets (T2), with T2 being much brighter than the initial target (T1) in the learning phase. The presentation of the relocated targets continued in a final transfer phase (10 blocks, epochs 9–10), but now the luminance of the target was again the same as in the learning phase. That is, in the final transfer phase, the target was presented at the relocated position (T2), not at its original position (T1). After each block, subjects took a short break and continued with the experiment at their own pace. In total, subjects completed 1224 trials. The overall experiment took about 1 h to complete.

#### Recognition test

After the search task, observers completed 24 trials, in which they had to decide via mouse button responses whether a particular display had been shown previously (old) or not (new). Old and new contexts were presented with the initial target locations because one would expect explicit recognition of a given old context (if existent at all) to be stronger for more reliably learned context-target relations. The response was non-speeded and no error feedback was provided.

### Results

#### Search task

Individual mean error rates were calculated for each variable combination. Overall, observers made relatively few errors (2.5%). A repeated-measures analysis of variance (ANOVA) with context (old, new) and Epoch (1–10) as within-subject factors was performed on the mean error rates. Note that Greenhouse–Geisser corrected values are reported in cases in which Mauchley’s test of sphericity was significant (*p* < 0.05). This analysis yielded a main effect of context, *F*(1, 17) = 4.9, *p* < 0.05, *η*^2^ = 0.22, but no other significant main or interaction effects (*p*s > 0.07). The context main effect showed that, somewhat more errors were made in new (2.7%) as compared to old (2.2%) context searches. This pattern of errors thus essentially corresponds to the results found for the analysis of the mean RTs (see below).

Next, individual mean RTs were calculated for old and new contexts separately for each epoch. Error trials and RTs faster than 300 ms and slower than 3000 ms were excluded from the analyses. This outlier criterion led to the removal of relatively few trials (0.5%); the same procedure also resulted in comparable exclusion rates in the subsequent experiments.

**Fig. 2:**
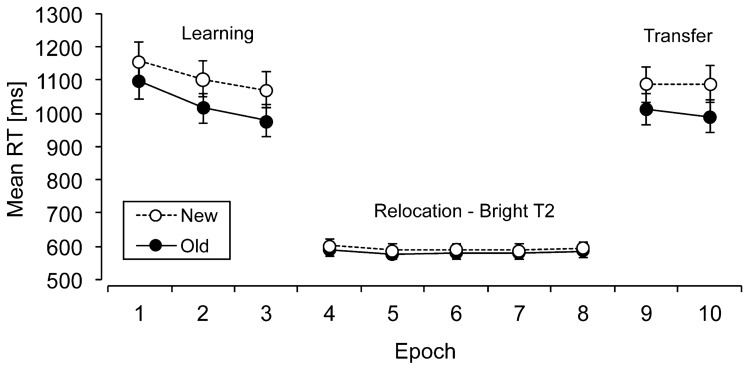
Mean reaction times (RTs, in milliseconds; with associated standard error bars) for old and new contexts (solid and dashed lines, respectively) as a function of epoch in Experiment 1

Individual mean RTs were computed for old and new contexts in each phase (learning, relocation, transfer) and epoch. Figure 2 presents RTs for old and new contexts across phases and epochs. An overall ANOVA with the factors Context (old, new) and Phase (learning, relocation, transfer) was performed to investigate whether contextual cueing changed in the different phases of the experiment. This analysis yielded significant main effects of context, *F*(1, 17) = 61.9, *p* < 0.001, *η*^2^ = 0.78, and of phase, *F*(1.33, 22.67) = 147.4, *p* < 0.001, *η*^2^ = 0.89, showing an overall contextual-cueing effect of 58 ms and overall faster RTs in the relocation phase (588 ms) as compared to the learning and transfer phases (1071 ms and 1045 ms, respectively, *p*’s < 0.001). There was also a significant interaction between context and phase, *F*(1.31, 22.21) = 5.46, *p* < 0.03, *η*^2^ = 0.24, which showed that contextual cueing was overall comparable in the learning (80 ms) and transfer (85 ms) phases (*p* = 0.87), but these cueing effects were (marginally) larger than in the relocation phase (11 ms, *p*’s < 0.06).

An additional analysis was also performed on all 32 observers (i.e., including the 14 observers that did not show an above-zero contextual-cueing effect in the learning phase). We have previously shown that observers who do not exhibit initial contextual learning tend to develop reliable contextual cueing in later parts of the experiment (Zellin et al., 2013a). Thus, the selection of observers with above-zero contextual cueing may lead to an “artificial” increase of the overall contextual-cueing effect during initial learning, but conversely, the complete sample of observers may in turn camouflage possible costs during adaptation due to some observers exhibiting a pattern of “late learning” (Zellin et al., 2013a). The 14 excluded observers in Experiment 1 also showed some evidence for late contextual learning with mean contextual-cueing effects of − 8 ms, 14 ms and 94 ms in the learning, relocation and transfer phases, respectively. The inclusion of these 14 late learners in the overall analysis (with now 32 observers in total) therefore reduced the initial cueing effect. However, the basic pattern of results nevertheless remained comparable: there were significant main effects of context, *F*(1, 31) = 21.55, *p* < 0.001, *η*^2^ = 0.41, and phase, *F*(1.36, 42.38) = 208.93, *p* < 0.001, *η*^2^ = 0.87, which again were due to an overall cueing effect of 37 ms and a reduction of the mean RTs during the relocation phase (583 ms) relative to the learning (1073 ms) and transfer (1028 ms) phases. Finally, the interaction was also significant, *F*(1.40, 43.49) = 8.79, *p* < 0.03, *η*^2^ = 0.22, which revealed a larger cueing effect of 90 ms in the transfer phase, as compared to the learning (9 ms, *p* = 0.006) and relocation (13 ms, *p* < 0.001) phases.

In a subsequent step, additional analyses were performed separately for each phase (on the sample of *N* = 18 learners). For the *learning phase* (initial target location, T1), an ANOVA with the factors Context (old, new) and Epoch (1–3) yielded significant main effects of context, *F*(1, 17) = 16.82, *p* < 0.002, *η*^2^ = 0.49, and of epoch, *F*(1.47, 25.04) = 20.41, *p* < 0.001, *η*^2^ = 0.54. RTs were on average 80 ms faster for old contexts as compared to new contexts and decreased by 103 ms across epochs. The interaction did not reach significance (*p* > 0.48), indicating that subjects already showed a robust contextual-cueing effect in epoch 1. To show that contextual cueing was due to actual learning and thus developed in the first epoch, additional pairwise comparisons between RTs for old and new contexts in the first ten blocks were computed, which revealed the first significant difference between RTs of old and new contexts already in block 3, *t*(17) = 2.46, *p* < 0.03, which is in line with previous reports of a quick onset of the contextual-cueing effect (e.g., Conci & von Mühlenen, 2009).

For the *relocation phase* (relocated target, T2), a further ANOVA with the factors Context (old, new) and Epoch (4–8) revealed a significant main effect of context, *F*(1, 17) = 5.36, *p* < 0.04, *η*^2^ = 0.24, which was due to a small (but reliable) RT benefit of 11 ms for old relative to new contexts. Thus contextual-cueing still remained efficient, even though the increase in brightness of the relocated target substantially expedited the overall search RTs (by 490 ms, see also Geyer et al., 2010). The main effect of epoch and the interaction between context and epoch were not significant (*p*s > 0.43). As in the learning phase, we again computed additional pairwise comparisons between RTs in old and new contexts across the first ten blocks to determine the point in time at which contextual cueing re-emerged after the change. These comparisons revealed the first significant difference to occur in the fourth block of the relocation phase, *t*(17) = 2.21, *p* < 0.05. Thus, the onset of relearning (in block 4 of the relocation phase) was roughly comparable to the onset of initial learning, which was evident from block 3 onwards.

**Fig. 3:**
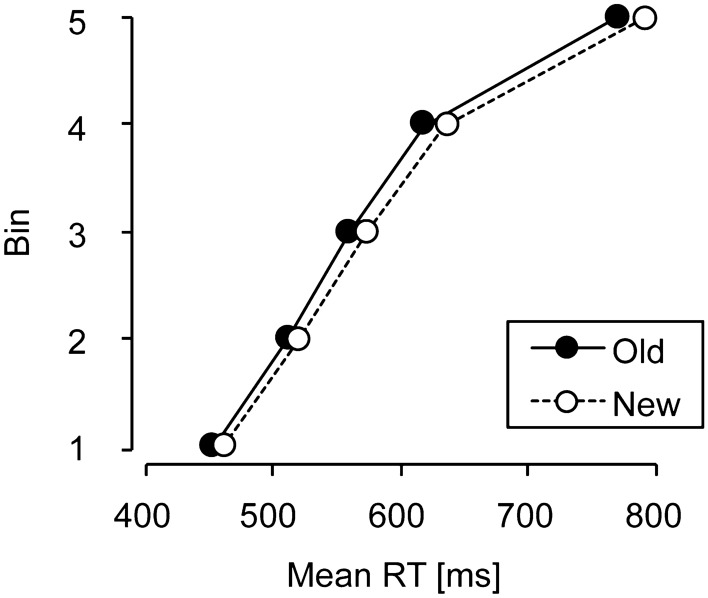
Vincentized, cumulative reaction time (RT) distributions for old and new contexts in Experiment 1 during the relocation phase, depicting a small but nevertheless reliable benefit for repeated, old context displays—as evidenced by a leftward shift of the entire RT distribution

Given the relatively small size of the cueing effect, the distribution of RTs in the relocation phase was further analysed to examine whether contextual cueing occurred throughout all trials, or only for a subset of responses (e.g., particularly for slow responses; see Johnson et al., 2007). To this end, individual RTs for old and new contexts in the relocation phase were ranked in ascending order, and quantiles (5 bins for each observer) were computed (vincentized RT distributions; Ratcliff, 1979) resulting in a group RT distribution (see Fig. 3). A repeated-measures ANOVA with the factors Context (old, new) and Bin (1–5) was computed and revealed significant main effects of context, *F*(1, 17) = 6.11, *p* < 0.03, *η*^2^ = 0.26, and bin, *F*(1.06, 18.03) = 149.47, *p* < 0.001, *η*^2^ = 0.89. Most importantly, the interaction between context and bin was not significant (*p* > 0.2), which means that contextual cueing occurred across faster as well as slower RTs, even when search was particularly fast overall. This indicates that contextual cueing shifted the entire RT distribution and not just a proportion of (e.g., relatively slow) responses.^1^

Finally, in the *transfer phase* (relocated target, T2), an ANOVA with the factors Context (old, new) and Epoch (9–10) revealed a significant main effect of context, *F*(1, 17) = 18.31, *p* < 0.002, *η*^2^ = 0.51, reflecting faster RTs for old contexts in comparison to new contexts (85 ms). This shows that relocated targets were successfully associated with old contexts even when the attention-guiding brightness signal was removed from the target. No further effects were significant (*p*s > 0.46).

#### Recognition test

The mean accuracy of recognizing old and new contexts was 50%. Categorization of old contexts as old (50.4% hits) did not differ significantly from identifying new contexts as old contexts (48.6% false alarms), *t*(17) =  − 0.63, *p* = 0.53. Thus, observers were not aware of the repeated displays, mirroring previous findings (Chun & Jiang, 1998).

### Discussion

In Experiment 1, we examined whether observers would be able to update contextual associations and incorporate the changed target locations, if the relocated target is rather salient (i.e., brighter) as compared to the surrounding context. We found that visual search for the bright, relocated target was overall much faster (by more than 400 ms) than search in the initial learning phase, thus showing that attention was successfully guided by the salient bottom-up signal. Interestingly, a small, but consistent, contextual-cueing effect was observed for the salient targets in the relocation phase (this benefit was also evidenced by a shift of the complete RT distribution). Moreover, the time course of relearning was found to mirror the time course of initial learning, with a contextual-cueing effect emerging quickly after only ~ 3 repetitions in both the learning and relocation phases. Contextual cueing also continued for relocated targets in the transfer phase when the brightness manipulation was removed and relocated targets were as salient as the other items in the display. This suggests that the bottom-up guidance of attention to changed target locations facilitated the updating of the previously learned contextual associations (to incorporate the changed target). This updating led to a persistence of the relearned contexts. Thus, the old contexts continued to guide visual search beyond the salience manipulation.

However, an alternative account to explain efficient contextual cueing for the relocated target would be related in particular to the salient brightness enhancement of the target. That is, the relocated target was not only salient, but it also predicted the target location (with 100% validity), thus possibly serving as an additional cue to facilitate relearning. However, it should be noted that the salient brightness enhancement was not specific to a particular (invariant) display, but instead the target-predictive brightness information was present in every single (old and new) display. The additional cue could therefore be exploited to facilitate learning in both old and new displays to a similar extent (because the enhanced brightness predicted the invariant target location in all displays, not just in the old layouts). Given this, the predictability of the brightness cue cannot explain why there was a benefit, which occurred in particular for the repeated (old) contexts. Rather, the results seem to be consistent with the above-mentioned account that assumes that the salience of the relocated target helps to overcome the attentional misguidance signal from the initially learned target location (see also Zinchenko et al., 2020).

Also, in general, *spatial* contextual cueing is usually not affected by changes of particular features in the search items themselves (e.g., Chun & Jiang, 1998; Ehringer & Brockmole, 2008), and adding a predictive feature to invariant search displays does usually not improve contextual learning (Conci & von Mühlenen, 2009; Kunar, John, & Sweetman, 2014). This indicates that the spatial configuration primarily reflects an effect of spatial layout rather than perceptual learning of the low-level image properties in the display.

Although the presentation of the salient, relocated target in the relocation phase was found to speed search quite considerably—and rendered learning of contextual cues rather redundant—observers nevertheless seemed to be additionally guided by the invariant contexts. This finding is consistent with previous reports that indicate that contextual cues still exert guidance on visual search despite reliable bottom-up signals (see also Geyer et al., 2010; Schankin & Schubö, 2010), that—in itself—may already provide optimal attentional guidance (Harris & Remington, 2017). Together, these results show that a selection bias due to acquired, contextual cues may influence performance in addition to a bias that arises from salient items that attract attention in a bottom-up manner. Thus, attention might be guided both by past experience and by salient information in the display.

## Experiment 2

To optimize visual search, attention may enhance processing of the target, but it may also inhibit concurrent (task-irrelevant) nontargets (Desimone & Duncan, 1995; Klein 1988). Moreover, statistical learning appears to increase these opposing biases even further. For instance, in contextual cueing, learning of repeated spatial layouts leads to an enhanced priority of the target, and conversely a decreased priority of the nontargets (Makovski & Jiang, 2010; Ogawa et al., 2007). The aim of Experiment 2 was to exploit the latter, inhibitory component of contextual cueing (relating to the nontargets) to potentially facilitate the updating of contextual memory after target relocation. Such an inhibitory signal was established in the relocation phase by placing a (to-be-inhibited) nontarget at the initial target location. In a final transfer phase, the relocated target was still presented, but the (inhibitory) nontarget was moved from the initial target location back to its original position (such that the initial target location was then empty, see Fig. 1b). If nontargets are indeed inhibited in contextual cueing, then moving a nontarget to the initial target location should therefore ease the adaptation towards the relocated target. That is, rather than facilitating relearning by providing a salient attention-guiding signal to the novel target location (as in Experiment 1), in Experiment 2, orienting towards the previously relevant target location was discouraged by presenting an inhibitory signal (i.e., a non-salient, but to-be-avoided nontarget L) at this location. Thus, the idea of this (essentially rather subtle) change in the display was that the presence of a nontarget at the previous target location should lead to inhibition at the initial target location, and this inhibitory signal should in turn facilitate the disengagement from that location. This should then ultimately trigger more flexible learning of the novel target location.

### Methods

Apparatus, stimuli, design, and procedure were similar to Experiment 1, except that all stimuli were always presented with the same luminance throughout the experiment, and instead, a nontarget was placed at the initial target location in the relocation phase. In the initial learning phase (epoch 1–3), (old and new) search layouts were presented with a first target location (T1) to establish a reliable contextual-cueing effect. Subsequently, the targets were presented at a novel, previously empty position (T2) in the relocation phase (epoch 4–8). Together with this relocated target, one nontarget from the eleven nontargets in a given display was randomly selected in each old-context layout and was placed at the initial target location of the respective context (see Fig. 1b)—the idea being that this nontarget would provide an inhibitory signal at that location (see e.g., Ogawa et al., 2007). Note, that a change to a single nontarget item should leave contextual cueing essentially unaffected (Conci & Müller, 2012), even though changes to nontargets that are closer to the actual target could be expected to reveal somewhat more interference with cueing than nontargets that are located further away (see Brady & Chun, 2007; Sisk, Remington, & Jiang, 2019). However, such a systematic bias is unlikely to influence the results given the random selection of the changed nontarget and the large variability of possible locations in the display. In the final transfer phase (epoch 9–10), relocated targets were still presented, but the initial target location was empty (i.e., the ‘inhibitory’ nontarget was again presented at the original position). In other words, during transfer, all nontarget positions were identical to the initial learning phase, while the target was presented at the changed location.

In Experiment 2, we again aimed to collect data from 18 “learners” (with an above-zero cueing effect in the learning phase). To achieve this target sample, the data from a total of 23 observers were collected. The final sample in Experiment 2 thus consisted of 18 adults (13 women, mean age: 22.3 years), and only 5 observers needed to be excluded. All observers reported normal or corrected-to-normal visual acuity and all but one observer were right-handed. They received either payment or course credits for their participation.

### Results

#### Search task

Individual mean error rates were calculated for each variable combination. Overall, observers made relatively few errors (2.8%). A repeated-measures ANOVA with context (old, new) and epoch (1–10) as within-subject factors revealed a significant main effect of epoch, *F*(3.55, 60.35) = 2.68, *p* < 0.05, *η*^2^ = 0.14, which indicated that errors increased to some extent with the duration of the experiment (i.e., there was a slight, linear increase by 2.1% from epochs 1 to 10). However, there were no significant main or interaction effects that included the factor context (*p*s > 0.08).

**Fig. 4:**
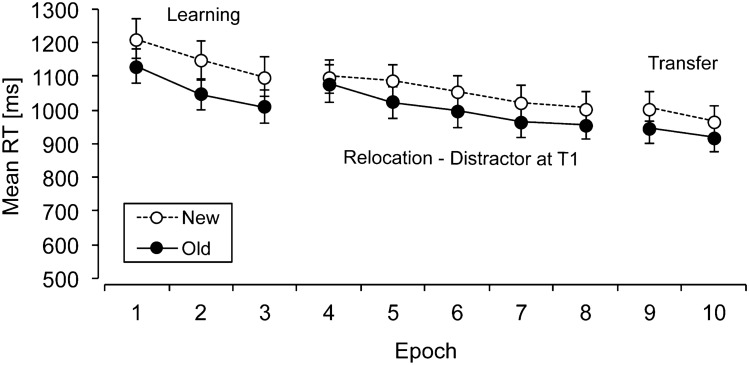
Mean reaction times (RTs, in milliseconds; with associated standard error bars) for old and new contexts (solid and dashed lines, respectively) as a function of epoch in Experiment 2

Next, individual mean RTs were computed for old and new contexts in each phase (learning, relocation, transfer) and epoch. Figure 4 shows the mean RTs for old and new contexts in each epoch (across phases). An overall ANOVA on the mean RTs with the factors context (old, new) and phase (learning, relocation, transfer) revealed significant main effects of context, *F*(1, 17) = 17.26, *p* < 0.002, *η*^2^ = 0.50, and of phase, *F*(2, 34) = 24.44, *p* < 0.001, η^2^ = 0.59, but no significant interaction between context and phase (*p* > 0.2). Thus, contextual cueing was not modulated to a significant extent across the experimental phases (see Fig. 4). Mean overall contextual cueing was 65 ms, and RTs were reduced by 147 ms from the initial learning to the final transfer phase.

Once again, we also performed an overall RT analysis on all 23 observers tested in Experiment 2. This analysis also showed significant context, *F*(1, 22) = 11.06, *p* < 0.004, *η*^2^ = 0.34, and phase, *F*(2, 44) = 23.06, *p* < 0.001, *η*^2^ = 0.51, main effects but no reliable interaction (*p* > 0.9). The mean overall contextual-cueing effect was 49 ms and RTs were speeded by 133 ms from the learning to the transfer phase. This shows that the results for all 23 observers were essentially comparable to the results obtained for our subsample of *N* = 18 “learners”. Next, separate analyses follow on the 18 “learners” to further explore the dynamics of contextual cueing in each phase.

In the *learning phase* (initial target location, T1), RTs were on average 90 ms faster for old contexts than for new contexts, and RTs decreased overall by 118 ms from epochs 1 to 3 (main effect of context, *F*(1, 17) = 29.99, *p* < 0.001, *η*^2^ = 0.64, main effect of epoch, *F*(1.27, 21.64) = 12.43, *p* < 0.01, *η*^2^ = 0.42, no interaction between context and epoch, *p* > 0.7). To demonstrate once again the development of contextual cueing, additional pairwise comparisons between RTs for old and new contexts were computed in the first ten blocks. As in Experiment 1, these comparisons again showed a rather quick onset of contextual cueing: the first significant difference between old and new context RTs was already evident in block 2, *t*(17) = 2.34, *p* < 0.04.

A comparable pattern again emerged in the *relocation phase* (relocated target, T2), which revealed a contextual-cueing effect of 49 ms and an overall RT reduction by 105 ms from epochs 4 to 8 (main effect of context, *F*(1, 17) = 4.47, *p* = 0.05, *η*^2^ = 0.21, main effect of epoch, *F*(4, 68) = 15.85, *p* < 0.001, *η*^2^ = 0.48, no interaction between context and epoch, *p* > 0.4). Additional pairwise comparisons between RTs in old and new contexts across the first ten blocks of the relocation phase were then again computed to determine when contextual cueing re-emerged after the change. For these comparisons, the first significant difference occurred somewhat later, in block 9 of the relocation phase, *t*(17) = 2.33, p < 0.04. Thus, effective relearning in Experiment 2 (from the ninth block of the relocation phase onwards) took somewhat longer than initial learning, which was as fast as in Experiment 1 and occurred already from block 2 onwards.

In the final *transfer phase* (relocated target, T2), mean contextual cueing for the relocated targets was again of comparable magnitude (56 ms) and there was a somewhat smaller reduction of the overall RTs of 34 ms from epochs 9 to 10 (main effect of context, *F*(1, 17) = 5.01, *p* < 0.04, *η*^2^ = 0.23, main effect of epoch, *F*(1, 17) = 6.56, *p* < 0.03, *η*^2^ = 0.28, no interaction between context and epoch, *p* > 0.4). Overall, this shows that contextual cueing was reliable both in the initial learning phase and after the target relocation.

#### Recognition test

Mean accuracy in the recognition test was 51%. The number of hits (52.2%) did not differ from the rate of false alarms (53.6%), *t*(17) = 0.55, *p* = 0.6, which indicates that observers were not aware of the display repetitions, mirroring the results of Experiment 1.

### Discussion

The results of Experiment 2 revealed a reliable contextual-cueing effect in all phases of the experiment, even though the target location changed from epoch 4 onwards. This indicates that the target location change was successfully incorporated into the previous context representation, which contrasts with findings from previous studies where the target location change was not accompanied by an additional inhibitory cue, and where the location change led to a substantial reduction of cueing (e.g., Zellin et al., 2013a; 2014). However, it should be noted that relearning was somewhat slower than initial learning (see the blockwise analysis above). Moreover, the overall cueing effect during the relocation phase was significant, yet (at least numerically) somewhat smaller than the cueing effect observed during initial learning (49 ms and 90 ms, respectively). Such a reduction might have occurred because cueing to a relocated target potentially incorporates associations to multiple (i.e., two) target locations (see also Conci et al., 2011 for a comparable, numerical reduction of cueing). These differences between learning and relearning might indicate that the updating of previously acquired contexts was indeed effortful. Nevertheless, the current results indicate that the inhibition of the initial target location (by a nontarget) was successful and facilitated relearning as reflected by robust contextual cueing in both the relocation and transfer phases. Since reliable contextual-cueing effects were found throughout both phases, the inhibition of the initially learned target location by the nontarget appears to have promoted a persistent updating of the changed contextual associations.

The results from Experiment 2 might also be relevant when considering an alternative account to explain efficient contextual cueing during the relocation phase. For instance, when considering the results from Experiment 1 alone, one might conclude that relearning was facilitated by the perceptual change that was inserted across phases in the target. That is, the change of the target (in terms of its brightness) might have caused some “reset” that could have promoted novel learning. Indeed, perceptual “resetting” might—to some extent—explain why relearning was faster in Experiment 1 (where a perceptual segmentation occurred between phases), as compared to Experiment 2 (where the target was perceptually always the same). However, Experiment 2 nevertheless revealed efficient relearning even though there was no perceptual segmentation between phases. Moreover, in a study by Conci et al. (2011) a non-salient perceptual change of the target identity (that occurred together with the target location change) did also not facilitate relearning. This suggests that attentional guidance rather than some form of perceptual “resetting” was the major driving factor to promote efficient relearning.

## Experiment 3

Experiments 1 and 2 demonstrated that both facilitatory and inhibitory attention-guiding signals can result in a more flexible and persistent updating of contextual cueing after a target location change. Experiment 3 was in turn performed to provide a baseline measure, to assess how contextual cueing would recover after a target location change when there is no attentional signal to facilitate the adaptation of the relocated target. To this end, an experimental setup was used that was identical to the previous two experiments (see Fig. 1c), except that there was no facilitatory (Experiment 1) or inhibitory (Experiment 2) bias that accompanied the target location change (in the relocation phase). That is, the experiment was essentially comparable to several previous studies, which showed that without additional guidance the updating of contextual cueing after a change is rather slow and inefficient (Manginelli & Pollmann, 2009; Zellin et al., 2013a; 2014).

### Methods

Apparatus, stimuli, design, and procedure were comparable to Experiment 1, except that all stimuli were always presented with the same luminance throughout the experiment. During an initial learning phase (epoch 1–3), old and new search contexts were presented with a first target location (T1) to establish a reliable contextual-cueing effect. Subsequently, the targets were presented at a novel, previously empty position (T2) in the relocation phase (epoch 4–10; see Fig. 1c). It should be noted that the results from this experiment were in part already presented in a previous study (Experiment 1B in Zellin et al., 2013a), where *N* = 12 observers with reliable (above-zero) contextual cueing were tested. To present a sample that consisted of 18 “learners” (as in Experiments 1 and 2 of the current study), 11 additional observers were tested. Five of these newly tested observers needed to be excluded (because they revealed a negative cueing effect during the initial learning phase), while the other six observers were included in the sample of learners. The final sample of learners in Experiment 3 thus consisted of 18 adults (11 women, mean age: 26.1 years). All observers reported normal or corrected-to-normal visual acuity and all were right-handed. They received either payment or course credits for their participation.

### Results

#### Search task

Individual mean error rates were calculated for each variable combination. Overall, relatively few errors (2.4%) were made, and a repeated-measures ANOVA with the within-subject factors context (old, new) and epoch (1–10) did not result in any significant main or interaction effects (*p*s > 0.25).

**Fig. 5:**
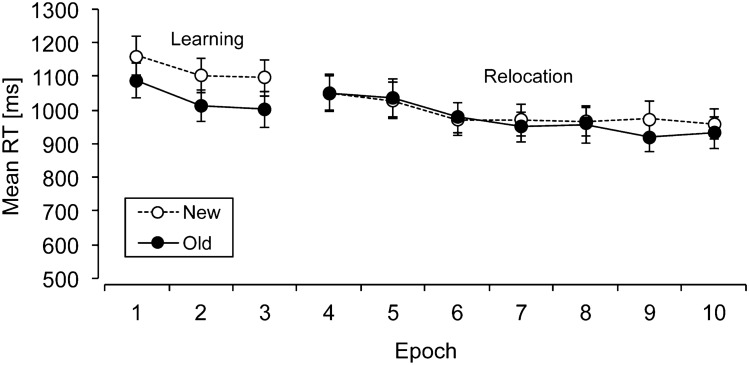
Mean reaction times (RTs, in milliseconds; with associated standard error bars) for old and new contexts (solid and dashed lines, respectively) as a function of epoch in Experiment 3

Next, individual mean RTs were computed for old and new contexts in the initial learning and in the subsequent relocation phase, separately for each epoch. Figure 5 shows the mean RTs for old and new contexts in each epoch (across phases). An overall ANOVA on the mean RTs with the factors context (old, new) and phase (learning, relocation) revealed significant main effects of context, *F*(1, 17) = 48.19, *p* < 0.001, *η*^2^ = 0.74, and of phase, *F*(1, 17) = 18.60, *p* < 0.001, *η*^2^ = 0.52, as well as a significant interaction between context and phase, *F*(1, 17) = 6.259, *p* < 0.03, *η*^2^ = 0.27. There was an overall contextual-cueing effect of 50 ms, and RTs were reduced by 96 ms from the initial learning to the subsequent relocation phase. Moreover, the significant interaction showed that contextual cueing was only evident in the initial learning phase (86 ms, *p* < 0.001), but not in the relocation phase (14 ms; *p* = 0.34).

We also performed an overall RT analysis on all 23 observers tested in Experiment 3 (i.e., also including the 5 observers who had to be excluded due to non-zero cueing effects). This analysis also showed significant context, *F*(1, 22) = 43.76, *p* < 0.001, *η*^2^ = 0.67, and phase, *F*(1, 22) = 25.13, *p* < 0.001, *η*^2^ = 0.53, main effects but no reliable interaction (*p* > 0.4). The mean overall contextual-cueing effect was 46 ms and RTs were 93 ms faster during relocation as compared to the initial learning phase. This shows that while contextual cueing was reliable in the whole sample, a reduction of cueing after the target location change was only evident when observers learned the initially presented invariant configurations (see also Zellin et al., 2013a). Next, separate analyses were performed on the 18 “learners” to further explore the dynamics of contextual cueing in each phase.

In the *learning phase* (initial target location, T1), RTs for old contexts were on average 86 ms faster than for new contexts, and across epochs 1–3 the mean RTs decreased overall by 77 ms (main effect of context, *F*(1, 17) = 25.26, *p* < 0.001, *η*^2^ = 0.59, main effect of epoch, *F*(1.44, 24.54) = 9.08, *p* < 0.004, *η*^2^ = 0.34, no interaction between context and epoch, *p* > 0.6). To again analyse the development of contextual cueing, additional pairwise comparisons between RTs for old and new contexts were computed in the first ten blocks. These comparisons revealed the first significant difference between RTs in old and new context in block 5, *t*(17) = 4.86, *p* < 0.001, thus once again showing that contextual cueing usually develops within the first epoch of a given experiment.

In the *relocation phase* (relocated target, T2), there was only a main effect of epoch, *F*(2.54, 43.26) = 12.87, *p* < 0.001, *η*^2^ = 0.43, which indicated that RTs decreased from epoch 4 (1052 ms) to epoch 10 (945 ms). However, there was no main effect of context (*p* = 0.34), nor an interaction between context and epoch (*p* > 0.08). Overall, this shows that contextual cueing was reliable in the initial learning phase but this benefit for old contexts vanished after the change of the target location, thus replicating previous findings (Manginelli & Pollmann, 2009; Zellin et al., 2013a; 2014).

#### Recognition test

Mean accuracy in the recognition test was 51%. The number of hits (58.3%) did not differ from the rate of false alarms (56.5%), *t*(17) = 0.43, *p* = 0.7, thus again indicating as in the previous experiments that observers were not aware of the display repetitions.

#### Cross-experiment comparison

A final analysis was performed to compare the cueing effects for the initial and relocated targets in Experiments 1 and 2 (which both provided a bottom-up guidance signal to facilitate the adaptation of the relocated target) to Experiment 3 (which served as a baseline and did not present any additional guidance signal in the relocation phase). To this end, the mean contextual-cueing effects in the learning and transfer phases of Experiments 1 and 2 were compared to the mean contextual-cueing effects in the learning and relocation phase of Experiment 3. Note that, using the data from the transfer phase in Experiments 1 and 2 ensured that all search displays were perceptually identical, that is, the comparisons could not be related to the additional guidance signal (that was presented in the relocation phase). As we had a-priori hypotheses about the direction of effects (we predicted that the guidance signal should improve contextual adaptation), one-tailed independent samples t-tests were used for post hoc comparisons.

First, an ANOVA which compared the cueing effects in the learning phase of all three experiments, showed no significant difference, *F*(2, 53) = 0.08, *p* = 0.92, *η*^2^ < 0.01: contextual cueing was overall comparable during initial learning across all three experiments (80 ms, 90 ms and 86 ms, in Experiments 1 through 3, respectively). In addition, a second ANOVA was performed to compare contextual cueing at the end of the learning phase (in epoch 3) with contextual cueing at the beginning of the relocation phase (in epoch 4) in the three experiments. This analysis showed that cueing was substantially reduced after the change of the target location (91 ms vs. 37 ms), *F*(1, 51) = 9.08, *p* < 0.005, *η*^2^ = 0.15, and this reduction after the change was statistically comparable across all three experiments—as evidenced by a non-significant epoch by experiment interaction (*p* = 0.093). Next, a third ANOVA, which compared contextual cueing for the (now) relocated targets showed a significant difference across the three experiments, *F*(2, 53) = 3.23, *p* < 0.05, *η*^2^ = 0.11. To determine the source of this significant difference, a series of posthoc comparisons were performed, which are theoretically of major interest to decide whether the additional guidance signals in Experiments 1 and 2 indeed facilitated relearning. These comparisons showed that contextual cueing during transfer was statistically comparable in Experiment 1 (85 ms) and Experiment 2 (54 ms), *t*(34) = 0.92, *p* = 0.18. However, contextual cueing during transfer in the two experiments with an additional guidance signal was significantly larger (mean cueing in Experiments 1 and 2: 69 ms) than cueing for the relocated target in Experiment 3 (14 ms), *t*(52) = 2.24, *p* < 0.015. Moreover, a significant difference was also revealed when comparing the relocated targets during transfer in Experiment 1 with Experiment 3, *t*(34) = 2.84, *p* < 0.005. Finally, cueing was also (marginally) larger in the transfer phase of Experiment 2 as compared to Experiment 3, *t*(34) = 1.42, *p* = 0.08.^2^ Thus, these analyses show (i) efficient learning initially and (ii) a substantial drop of learning after the target location change in all three experiments, whereas (iii) the updating of a previously acquired contextual memory was indeed facilitated by bottom-up guidance signals (in Experiments 1 and 2). This support of memory updating by means of guidance signals was particularly successful in Experiment 1, where the salience of the relocated target was increased.

### Discussion

Experiment 3 served to establish a baseline measure to determine memory updating in contextual cueing subsequent to a target location change when there is no accompanying attentional bias to facilitate the adaptation of the relocated target. The results revealed a reliable contextual-cueing effect of 86 ms in the initial learning phase of the experiment, but after the target location change, no reliable contextual-cueing effect (14 ms) was evident in epochs 4–10. This shows that contextual cueing essentially vanished after the target location change, without revealing any major evidence for a recovery of the learned representations across the entire relocation phase, thus replicating several previous findings (e.g., Manginelli & Pollmann, 2009; Zellin et al., 2013a; 2014). Additional cross-experiment comparisons indicated that this pattern of results also contrasted with the findings from Experiments 1 and 2, where the changed target location was successfully incorporated into the previous context representation. Thus, contextual memory updating can be “helped” by providing a facilitatory, or an inhibitory attentional guidance signal that accompanies the change of the target.

## General discussion

This study investigated whether bottom-up attentional (facilitatory and inhibitory) biases can influence the updating of context memories subsequent to an environmental change (that is, a change of the target location within an otherwise invariant spatial context). Previous studies consistently found that a change of the target leads to a substantial reduction in contextual cueing (of around 97% as indicated in a recent meta-analysis by Annac et al., 2017). In contrast to these previous findings, we found that adaptation to changed target locations was successfully achieved—as reflected by robust contextual-cueing effects both before and after the change. This shows that the stimulus-driven guidance of attention towards a changed target location (Experiment 1) and the diversion of attention away from an initially learned (but later no longer relevant) target location (Experiment 2) can indeed enhance the flexibility of contextual cueing. Conversely, a final control experiment once again replicated previous findings and showed that without any additional guidance, the updating of contextual cueing after a change is inefficient and slow (Experiment 3).

In our experiments, invariant spatial contexts were paired with unique target locations and repeatedly presented in an initial learning phase, during which robust contextual cueing was revealed (in the majority of our observers). Then, targets were relocated within their contexts and remained at their new locations for all subsequent presentations. When relocated targets were transiently made more salient than the surrounding spatial context of nontargets (Experiment 1), visual search was overall significantly speeded by bottom-up attentional guidance. Despite fast and efficient search, we also observed a small but reliable contextual-cueing effect for the relocated, salient targets. This cueing effect for the relocated target persisted beyond the transient saliency manipulation (i.e., it was still evident in the final transfer phase), and the time course of learning (after the change) was comparable to initial learning.

In Experiment 2, the inhibition of old context-target associations also facilitated adaptation to change: contextual cueing occurred for the relocated targets when initial target locations were de-prioritized by presenting an inhibitory (to-be-avoided) nontarget at the previous target position (Klein, 1988; see also Makovski & Jiang, 2010; Ogawa et al., 2007). Contextual cueing for relocated targets also continued to facilitate search after the inhibiting nontarget was moved back to its original position (transfer phase). Again, the benefit of the inhibitory nontarget led to effective contextual adaptation. Thus, contextual representations were successfully updated during the relocation phase, revealing memory-based guidance towards the changed target location. However, it should be noted that the recovery of learning after the change was somewhat slower than initial learning, and the cueing effect in the relocation phase was also numerically a bit smaller than cueing during initial learning. This might be taken to indicate that contextual updating is more effortful with additional inhibitory cues (Experiment 2) as compared to facilitatory cues (as used in Experiment 1). Nevertheless, both facilitatory and inhibitory attentional biases enhanced the updating of contextual cueing after a change, as compared to a control experiment that did not present any accompanying, additional cue with the target location change (Experiment 3). This indicates that the (bottom-up) modulation of attentional guidance can facilitate the updating of a given memory representation—thereby permitting an enduring adaptation to change in contextual learning.

Together, the current results show that stimulus-driven attentional guidance can increase the relocated targets’ priority, e.g., on a spatial map that represents a given invariant spatial layout. In a typical model of contextual cueing, search displays are represented as spatial maps that code the items in a given display (Brady & Chun, 2007; Zellin et al., 2013b). In the beginning of an experiment, search items in the displays receive overall roughly equal levels of activation. The repeated presentation of some invariant display layouts (throughout the experiment) in turn leads to the build-up of associations between the target and the surrounding nontarget items, and this learning in turn increases the activation of the target location in a given old display, thus revealing the typical contextual-cueing effect. In this view, the target locations in old, repeated displays leads to an increase in priority through contextual learning (see also Geyer et al., 2010).

Subsequent to an unexpected change of the target location, the activations on the attention-guiding map are no longer helpful for search but they instead provide a persistent misguidance signal (Zinchenko et al., 2020). As a result of this, target locations no longer benefit from their learned contextual associations. Instead, contextual cueing is substantially reduced when targets are suddenly relocated to new positions (e.g., Manginelli & Pollmann, 2009; see also Experiment 3), and successful contextual adaptation to the new target locations typically requires time-consuming, rather effortful training to overcome the initially acquired bias (e.g., 80 repetitions of the relocated targets, see Zellin et al., 2014). In contrast, in the current study, the location change was incorporated into the context memory representation *relatively* quickly (after some 4–9 repetitions of the relocated target)—because the changed targets were additionally prioritized by the bottom-up guidance manipulation. Our current results thus show that adaptation is significantly speeded if attention is guided to changed target locations (Experiment 1), or if attention is guided away from initial target locations (Experiment 2). Presumably, such a modulation of attention is facilitated by an adjustment of priority in the search-guiding spatial map.

While the present results indicate that implicitly acquired contextual cues guide attention in visual search, they also show that such involuntary learning requires selective attention in the first place to achieve memory-based guidance subsequently. For instance, guiding attention incidentally towards the relocated target facilitated the updating of the previously acquired contextual associations. It thus appears that flexible contextual cueing in fact depends on the attentional engagement towards relevant target-context associations (Jiang & Chun, 2001; Beesley, Hanafi, Vadillo, Shanks, & Livesey, 2018). Conversely, being distracted from the search context reduces the size of contextual cueing (Conci & von Mühlenen, 2009). At the same time, contextual cueing has also shown to reveal a performance benefit even when attentional guidance is already optimal (Harris and Remington 2017) or when a spatial cue is provided in addition to the contextual cue (Schankin & Schubö, 2010). Thus, a selection bias derived from an invariant spatial layout may influence performance in addition to a bias that arises from bottom-up salience computations. Attention might therefore be guided both by past experience and by salient information in the display.

In more general terms, contextual cueing appears to reflect processes of attentional guidance on the basis of past experience; it comes about as a result of the registration of invariant spatial regularities in the environment and thus neither reflects purely goal-directed nor stimulus-driven attentional biases, but rather some bias that has been referred to as “derived” attention (Le Pelley, Mitchell, Beesley, George, & Wills, 2016). The current study shows that such an acquired, i.e., learned, attentional bias can be modulated by stimulus-driven factors.

In sum, the present study reveals that implicit memory representations of spatial contexts are adapted to changes in the learned input rather quickly and reliably, if attention is either directed to the changes or if old associations are inhibited. This suggests that the influence of a given contextual cue on visual search can be modified by redirecting attention away from these associations.

## Footnotes

1 We would like to thank Torsten Schubert for suggesting this RT distribution analysis.

2 It should be noted that this comparison would not be significant when applying a Bonferroni correction to control for the family-wise error rate.
